# Constructing a Novel Prognostic Signature Based on TGF-*β* Signaling for Personalized Treatment in Pancreatic Adenocarcinoma

**DOI:** 10.1155/2022/4419119

**Published:** 2022-09-16

**Authors:** Tengfei Ji, Hongsheng Wu, Keqiang Ma

**Affiliations:** Department of Hepatobiliary Surgery, Affiliated Huadu Hospital, Southern Medical University (People's Hospital of Huadu District), Guangzhou 510800, China

## Abstract

**Background:**

Pancreatic adenocarcinoma (PAAD) shows significantly high mortality. Transforming growth factor-beta (TGF-*β*) signaling plays an important role in tumorigenesis and development. A prognostic model was conducted using transforming growth factor-beta (TGF-*β*) signaling for predicting PAAD prognosis and guiding personalized therapies.

**Methods:**

Datasets were grouped into test and training sets. Univariate Cox regression analysis and least absolute shrinkage and selection operator (LASSO) were applied and introduced for identifying prognostic genes associated with TGF-*β*. Risk score of each sample was calculated by the prognostic model. The difference in survival, clinical information, mutations, pathways, and chemotherapy and immunotherapy sensitivities between high-risk and low-risk groups was analyzed.

**Results:**

Based on TGF-*β* signaling, this work built a 7-gene prognostic model showing robustness in sample classification into low-risk and high-risk groups with differential prognoses. Oncogenic pathways like glycolysis, Notch signaling, and hypoxia were noticeably enriched in the group with high risk. Interferon and STAT1 were positively associated with risk score. Importantly, the low-risk group may develop a more favorable response to both chemotherapy and immunotherapy. The current work highlighted the significant function of TGF-*β* signaling in PAAD development and described the potential cross-links with other oncogenic pathways.

**Conclusion:**

Notably, the prognostic signature can act as a predictor of prognosis, but as a biomarker for optimizing personalized therapies in clinical practice.

## 1. Introduction

In 2020, an estimated number of 495,773 new cases of pancreatic adenocarcinoma (PAAD) were diagnosed according to global cancer statistics [1]. Compared to other cancer types, PAAD-affected patients consist of a relatively small percentage (2.6% in all cancers); however, the number of new deaths in 2020 is almost near its new affected individuals (466,003 deaths and 4.7% in all deaths). PAAD has extremely high mortality and is a very challenging cancer type in clinical treatment. A guideline proposed by National Comprehensive Cancer Network (NCCN) suggests various strategies for treating PAAD with different stages and conditions [2]. Curative resection is a major treatment for PAAD without distant metastasis, and its 5-year survival is estimated to be 27% [3]. However, still, a high rate of deaths is presented every year. Over the past two decades, the 5-year survival of metastatic PAAD is only improved from 1% to 2% [3]. Although chemotherapy such as fluorouracil, irinotecan, and oxaliplatin is a common strategy for treating metastatic patients, still a high recurrence causes high mortality.

Immunotherapy is a hopeful way for personalized treatment in difficult-to-treat cancers and has been steadily developing in recent years. Particularly, immune checkpoint blockade, for instance, cytotoxic T lymphocyte-associated protein 4 (CTLA-4) and programmed cell death protein 1 (PD-1) inhibitors achieve positive progress in treating some metastatic cancer types, although many are still under clinical trials [4]. In the exploration of immunotherapy, tumor microenvironment (TME) will influence the design or efficiency of immunotherapy. From pan-cancer research, based on five aspects including wound healing, inflammatory, lymphocyte infiltration, macrophages, and TGF-*β* response, IFN-*γ* response, cancers were classified into six immune subtypes (C6 to C1) [5]. PAAD is classified into four major immune subtypes, C6 (TGF-*β* Dominant), C3 (Inflammatory), C2 (IFN-*γ* Dominant), and C1 (Wound Healing) [5]. These immune subtypes provide guidance for facilitating the development of personalized therapy.

Previous studies have demonstrated that the TGF-*β* signaling pathway plays a central role in cancer progression and metastasis [6, 7]. TGF-*β* is a cytokine critical for modulating fibrotic response, extracellular matrix, epithelial-mesenchymal transition (EMT), and TME in cancer [6, 8]. In pancreatic cancer, TGF-*β* is obviously overexpressed, and the TGF-*β* signaling pathway is one of the critical oncogenic pathways involved in cancer progression. Most PAAD patients occur mutations in TGF-*β* signaling-related genes [9]. TGF-*β* signaling pathway has dual functions with cancer suppression in the early stage and cancer promotion in the late stage [10]. This characteristic makes it a challenging task to target the TGF-*β* signaling pathway for developing targeted drugs of PAAD.

Up to now, various biomarkers for predicting PAAD prognosis and TGF-*β* inhibitors for suppressing PAAD have been explored under preclinical studies [10]. To further understand the interaction among TME, cancer development, and TGF-*β*, we considered to construct a prognostic model based on the TGF-*β* signaling pathway for guiding the targeted therapy. Here, TGF-*β*-associated genes were included for identifying prognostic genes and characterizing the relation between TGF-*β* and immune response. The prognostic signature developed here in this work was predictive of PAAD prognosis and provided a direction for chemotherapy and immunotherapy.

## 2. Materials and Methods

### 2.1. Data Information

**Figure 1:**
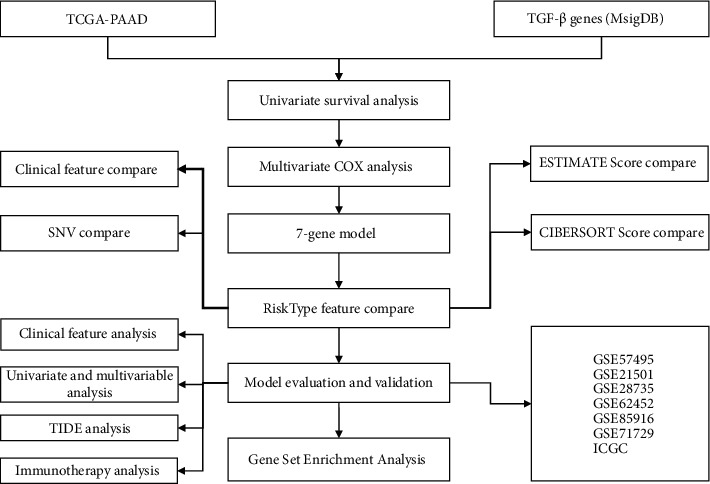
The workflow of constructing a prognostic model for pancreatic adenocarcinoma.

TCGA-PAAD dataset including RNA-seq data and clinical information was acquired from the TCGA database on June 30, 2021. From ICGC, the PACA-AU dataset (described as ICGC in the following text) including expression profiles and survival data was downloaded on June 30, 2021. GSE cohorts (GSE57495, GSE21501, GSE28735, GSE62452, GSE85916, and GSE71729) containing survival data and expression profiles were downloaded from GEO on June 30, 2021. From Molecular Signature Database (MSigDB, v7.4, the TGF-*β* pathway was obtained and 54 genes in total associated with TGF-*β* were included. The workflow *o* was shown in Figure 1.

### 2.2. Data Preprocessing

In the TCGA-PAAD dataset, samples without survival time and status or follow-up information were eliminated. Ensembl ID was transformed into a gene symbol. When multiple gene symbols to one gene, median expression was selected. In GSE cohorts, samples without time and status were eliminated. The probes were transformed into gene symbols. If a probe corresponded to multiple genes, it was then eliminated. Median expression was selected if one gene with multiple gene symbols. After data preprocessing, the information of samples in eight datasets was displayed (Supplementary Table S1).

### 2.3. Univariate and Multivariate Cox Regression Analysis

Univariate and multivariate Cox regression analyses are common methods for identifying risk factors for survival. Here, univariate Cox regression analysis screened 27 TGF-*β*-associated genes with *P* < 0.05 and hazard ratio (HR) > 1. Through univariate and multivariate Cox regression analysis, clinical features such as ages, genders, and stages were included to evaluate the independence of risk score as a risk factor for PAAD.

### 2.4. Least Absolute Shrinkage and Selection Operator (LASSO) Analysis

LASSO is a method to simplify variable numbers by using a penalty function for constructing an optimal model. Glmnet R package was used to conduct LASSO regression [11]. Lambda value is introduced in the progress of optimizing a model. The coefficients close to zero showed an increased lambda value. To assess models in different lambda values, 10-fold cross-validation was used. Optimal lambda was chosen under the glmnet algorithms for determining the variables. The prognostic model was determined with the formula

(1) $$\begin{array}{c} \text{risk score}=\text{coefficient}1*\text{gene expression}1+\text{coefficient}2*\text{gene expression}2\\ +\cdots +\text{coefficientn}*\text{gene expression}n\text{.} \end{array}$$

### 2.5. Definition of Low-Risk and High-Risk Groups

Sample risk score was determined in one dataset by the prognostic signature. For determining the optimal cut-off of the risk score in sample classification into two risk groups (high and low), the survminer R package was performed. Kaplan-Meier survival was introduced in the survival probability. AUC and ROC curves were used to evaluate the prediction efficiency of 5-year, 3-year, and 1-year survival for the prognostic model through the timeROC package [12].

### 2.6. Mutation Analysis

Tumor mutation burden (TMB) and mutation patterns of high-risk and low-risk groups were assessed. The mutation data of the TCGA-PAAD dataset downloaded from TCGA was already processed by the Mutect2 tool. Mutect2 is a popular method by using a Bayesian classifier for filtering mutations with high specificity and sensitivity and is commonly used for pan-cancer research [13].

### 2.7. SsGSEA and GSVA

GSVA R package was employed to conduct ssGSEA in assessing enrichment pathways [14]. GSEA allows to define sample enrichment scores in a gene set, which can indicate absolute enrichment of a gene set [15]. In analyzing enriched pathways of low-risk and high-risk groups, hallmark pathways include a series of gene sets (h.all.v7.4.symbols.gmt) from MSigDB. Expression data of TCGA-PAAD was used as input to calculate the enrichment score of pathways for each sample. *P* < 0.05 was the threshold to output significantly enriched pathways. GSVA is an unsupervised and nonparametric method to calculate enrichment scores inside and outside a gene set [14]. GSVA was performed to analyze the enrichment of 7 inflammatory metagenes (HCK, LCK, MHC I, MHC II, IgG, and STAT1) in high-risk and low-risk groups.

### 2.8. Functional Analysis

WebGestaltR (version 0.4.4) package [16] was applied to assess GO and KEGG pathways in TCGA-PAAD dataset. Cellular components, molecular functions, and biological processes are included in GO. WebGestalt is a popular tool for the annotation of interested genes based on a list of functional categories. *P* < 0.05 filtered significantly enriched terms, and we visualized the top 10 enriched terms.

### 2.9. Assessment of Immune Microenvironment

ESTIMATE R package was used to calculate the stromal score and immune score [17], and the ESTIMATE score is generated from the combined stromal and immune score. ESTIMATE is convenient to describe immune infiltration of tumor tissue through expression profiles.

CIBERSORT is also a measurement for characterizing tumor immune infiltration based on expression data [18]. It calculates the enrichment score of immune cells within 22 cell types. CIBERSORT introduced a machine learning approach that can improve the accuracy of resolving cell subsets in mixtures.

### 2.10. Prediction of Efficacy of Immunotherapy and Chemotherapy

For predicting the sensitivity to immunotherapy, we implemented SubMap analysis through comparing the similarity of expression data between two datasets [19]. To this end, we obtained the IMvigor210 dataset consisting of urothelial carcinoma samples treated with anti-PD-L1 [20]. The expression data of TCGA-PAAD and IMvigor210 datasets were compared grouped by stable/progressive disease and complete/response partial response. Bonferroni-corrected *P* < 0.05 was considered as a significant similarity. As for the sensitivity prediction to chemotherapy, the estimated IC50 (biochemical half maximal inhibitory concentration) of five drugs including cisplatin, erlotinib, sorafenib, paclitaxel, and crizotinib was calculated using the pRRophetic R package [21].

### 2.11. Statistical Analysis

All statistical analysis was conducted in the R (v3.4.2) platform. The statistical method was indicated in the corresponding legends. Bonferroni correction was used to correct the *P* value. Significance was considered if *P* < 0.05. ^*∗∗∗∗*^*P* < 0.0001, ^*∗∗∗*^*P* < 0.001, ^*∗*^*P* < 0.05, ^*∗∗*^*P* < 0.01. ns, no significance.

## 3. Results

### 3.1. A Prognostic Model Based on TGF-*β*-Associated Genes for PAAD

**Figure 2:**
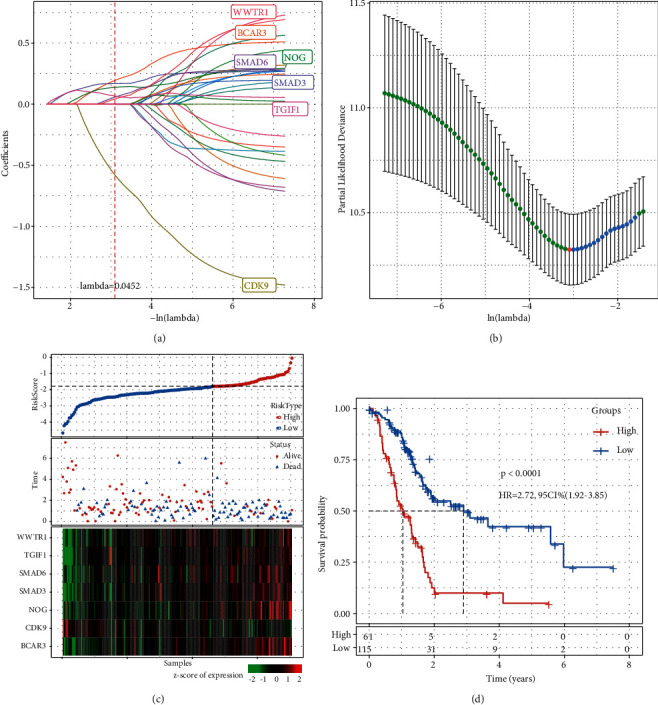
Construction of a 7-gene signature in TCGA-PAAD dataset. (a) The trajectory of each gene (variable) with the changing lambda. Vertical axis indicates the coefficients of each gene, and horizontal axis indicates the lambda value shown as–ln(lambda). Red dotted line represents the position of lambda = 0.0452. (b) The confidence interval of the changing lambda. Red dot corresponds to the red dotted line. (c) The survival status and expression of 7 prognostic genes of 176 samples ranking by risk score. Risk score and mRNA expression were converted to z-score. (d) Kaplan-Meier survival plot of high-risk and low-risk groups. Log-rank test was performed. HR, hazard ratio. CI, confidence interval.

First, we searched TGF-*β* signaling pathway from MSigDB and obtained 54 genes associated with TGF-*β*. In the TCGA-PAAD dataset, univariate Cox regression analysis was used to screen genes related to overall survival, and 28 genes were identified where 27 genes were risk factors (*P* < 0.05, HR > 1). Then LASSO cox regression was applied to decrease the number of screened genes and construct a prognostic model. With the increasing lambda value, the coefficient of each gene was close to zero, resulting in a decreased number of variables (Figure 2(a)). When lambda = 0.0452, the optimal model with the least number of genes (variables) was presented (Figure 2). Using multiple cox regression analyses for the remaining 7 genes, we obtained their coefficients, the following formula was used to define the 7-gene prognostic model:

(2) $$\begin{array}{c} \text{Risk score}=0.186*\text{SMAD}6+0.174*\text{SMAD}3+0.16*\text{WWTR}1\\ +0.026*\text{TGIF}1-1.021*\text{CDK}9+0.151*\text{NOG}+0.334*\text{BCAR}3. \end{array}$$

We calculated the sample risk score in the TCGA-LAAD dataset, and delineated the distribution of samples from low-risk scores to high-risk scores (Figure 2(c)). For classifying samples into high-risk and low-risk groups, the survminer R package was used to select the optimal cut-off. Dead samples were highly accumulated in the high-risk group. Excluding *CDK9*, the other six genes (*SMAD6*, *SMAD3*, *WWTR1*, *TGIF1*, *NOG,* and *BCAR3*) in the high-risk group were higher expressed. Kaplan–Meier survival analysis manifested a significant difference in survival between the two groups, with 61 and 115 samples and classified into two risk groups, respectively (*P* < 0.00001, HR = 2.72 (95% CI: 1.92–3.85), Figure 2(d)). We used ROC to evaluate the predicting effectiveness of 5-year 3-year and 1-year, and the result presented a high AUC of 0.74, 0.76, and 0.81, respectively (Supplementary Figure S1A).

**Figure 3:**
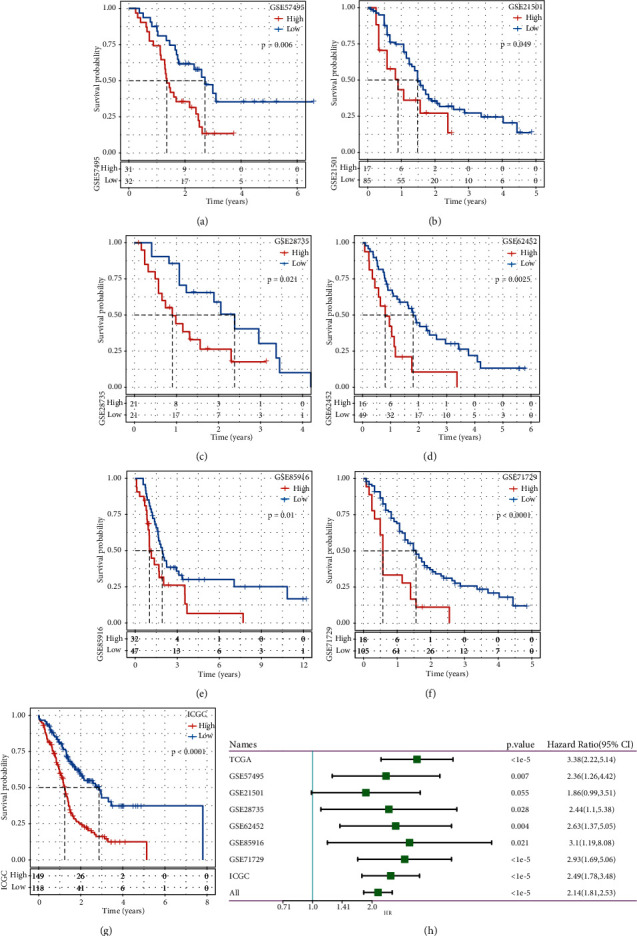
(a–g) Kaplan-Meier survival plot of independent datasets (GSE57495, GSE21501, GSE28735, GSE62452, GSE85916, GSE71729 and ICGC). (h) Univariate cox regression analysis of risk score in all eight datasets. Log-rank test was performed. HR, hazard ratio. CI, confidence interval.

Then seven independent datasets (GSE57495, GSE21501, GSE28735, GSE62452, GSE85916, GSE71729, and ICGC) were used to validate the prognostic model. By using the same analysis, we calculated the risk score of each sample according to the expression of seven prognostic genes. Kaplan-Meier survival plots of seven datasets were described, and these samples were significantly classified into two groups (*P* < 0.05, Figures 3(a)–3(g)). ROC analysis exhibited favorable AUC of 1-year, 3-year, and 5-year in these datasets except for GSE21501 having relatively low AUC (Supplementary Figures S1B–S1H). Univariate Cox regression analysis revealed a high HR in the high-risk group (*P* < 1*e* − 5, all HR = 2.14 (95%CI: 1.81–2.53), Figure 3(h)), indicating that this prognostic model was effective to predict prognosis and TGF-*β* signaling pathway was a risk factor to PAAD patients.

### 3.2. Mutation Characteristics of High- and Low-Risk Groups

**Figure 4:**
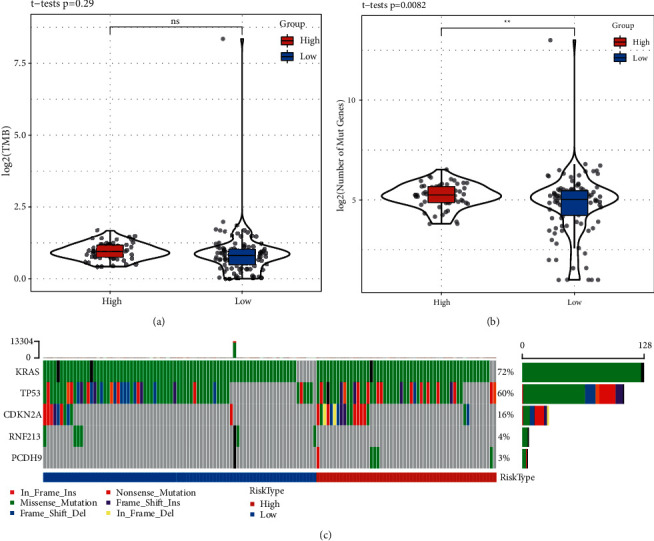
Mutation features of high-risk and low-risk groups. (a and b) Comparison of TMB (a) and the number of mutated genes (b) in high-risk and low-risk groups. Student *t* test was performed. (c) Mutation patterns of significantly mutated genes in high-risk and low-risk groups. TMB, tumor mutation burden. Ns, no significance, ^*∗∗*^*P* < 0.01.

Mutation data in the TCGA-PAAD dataset was used to analyze TMB and mutation patterns through mutect2 software. No significant difference in TMB was observed in high- and low-risk groups (*P*=0.29, Figure 4(a)), but the number of mutations exhibited differentially between the two groups (*P*=0.0082, Figure 4(b)). Furthermore, genes with a mutated frequency >3% were screened and a *Chi*-square test was performed to screen significantly mutated genes (*P* < 0.05). Finally, five genes (*KRAS*, *TP53*, *CDKN2A*, *RNF213,* and *PCDH9*) were identified, and their mutation patterns in high- and low-risk groups were both presented (Figure 4(c)). *KRAS* and *TP53* contributed the majority of mutations with a mutated frequency of 72% and 60% respectively, and they were commonly reported in various cancers. Missense mutation type consisted of almost mutations in *KRAS*, while *TP53* presented abundant mutation types such as nonsense mutation and frame-shift insertions or deletions.

### 3.3. Risk Score Is Associated with Clinical Features

**Figure 5:**
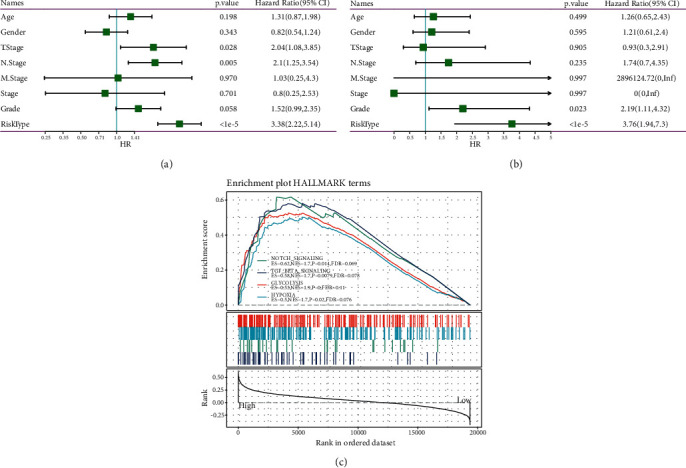
Univariate (a) and multivariate (b) cox regression analysis of the 7-gene signature and other clinical features. HR, hazard ratio. CI, confidence interval. (c) Gene set enrichment analysis of hallmark pathways of each sample ranking by risk score in TCGA-PAAD dataset. ES, enrichment score. NES, normalized enrichment score. FDR, false discovery rate.

The relationship between risk score and clinical features including gender, age, N stage, stages I to IV, T stage, grade, and M stage was assessed. There was no significant distribution difference in risk scores in different stages, genders, and ages (*P* > 0.05), except for grades G1 to G4 (*P* < 0.05, Supplementary Figure S2). However, we found that the 7-gene signature could effectively divide samples with different clinical features into high- and low-risk groups (*P* < 0.01, Supplementary Figure S3), suggesting that the prognostic model was valid in predicting overall survival in different clinical features. To demonstrate the advantage of the prognostic model, we compared it with other clinical features using both univariate and multiple Cox regression analysis. T stage and risk type, N stage as risk factors were all significantly associated with prognosis, as shown by Univariate Cox regression analysis (*P* < 0.05, Figure 5(a)), but risk type had the highest HR (3.38, 95%CI: 2.22–5.14). Multiple Cox regression analysis also revealed that risk type was the most associated with prognosis (*P* < 1*e* − 5, HR = 3.76, 95%CI: 1.94–7.3, Figure 5(b)).From these results, the 7-gene signature has been proven to be sufficient and robust to be applied as a prognostic predictor for patients with PAAD.

### 3.4. Functional Pathways Related to Risk Score

To analyze the enrichment of pathways in high- and low-risk groups, we selected a gene set of h.all.v7.4.symbols.gmt involved in hallmark pathways. By using the expression profiles of the TCGA dataset as an input in GSEA, the enrichment score of each sample was generated and ranked by the risk score. Four oncogenic pathways including TGF-*β* signaling, hypoxia, glycolysis, and notch signaling pathways were significantly enriched, with high enrichment in high-risk group (*P* < 0.05, Figure 5(c)). In addition, we selected 177 genes significantly associated with risk score (|*R*| > 0.6, *P* < 0.05), and most genes were positively associated with risk score (Supplementary Figure S4A). Then, we employed the WebGestaltR package to annotate these genes in GO terms and KEGG pathways. As a result, in GO terms, 16 molecular function terms, 33 cellular component terms, and 126 biological process terms were annotated (FDR < 0.05, Supplementary Figures S4B–S4D). 34 KEGG pathways with some related to cancer were significantly enriched in these genes including focal adhesion, ECM-receptor interaction, small cell lung cancer, PI3K-Akt signaling pathway, and proteoglycans in cancer (FDR < 0.05, Supplementary Figure S4E). The above results suggested that TGF-*β*-associated genes were strongly involved in cancer development.

### 3.5. The Relation between Risk Score and Immune Response

**Figure 6:**
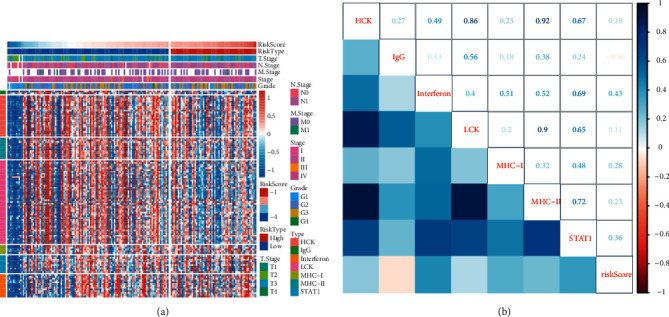
The relation between inflammatory-related metagenes and risk score in TCGA-PAAD dataset. (a) Heatmap of the metagene expression ranking by risk score. Red indicates relatively high expression and blue indicates relatively low expression. (b) Pearson correlation analysis between enrichment score of seven metagenes and risk score. Blue indicates positive correlation and red indicates negative correlation. Correlation coefficients were presented in the corresponding positions.

To understand the associated risk score with immune infiltration, we applied ESTIMATE and CIBERSORT methods to evaluate the immune infiltration in high- and low-risk groups. Using ESTIMATE measurement, we calculate stromal score, immune score, and ESTIMATE score. We observed that the low-risk group had higher scores on three terms, and no significant difference was found between the two groups (*P* > 0.05, Supplementary Figure S5). CIBERSORT analysis on 22 immune cells revealed that the low-risk group showed a higher enrichment of CD8 T cells, but a lower proportion of M0 macrophages, and M1 macrophages than the high-risk group (*P* < 0.05, Supplementary Figure S5D). Furthermore, we obtained 7 immune-related metagenes from previous research including IgG, interferon, MHC-II, LCK, STAT1, MHC-I, and HCK [22]. The enrichment score of 7 metagenes in each sample was calculated by GSVA. The expression heatmap of each sample in the TCGA-LAAD dataset was presented by risk score from low to high (Figure 6(a)). A low expression level of 7 metagenes was obviously shown in an extremely low-risk score. To further know the correlation between risk score and 7 metagenes, we performed the Pearson correlation analysis and found that most metagenes were positively correlated with risk score excluding IgG (Figure 6(b)). Interferon and STAT1 had relatively high correlation coefficients with 0.43 and 0.36, respectively, indicating that gene sets in the two metagenes had a close interaction with risk score or TGF-*β*-associated genes.

### 3.6. Predicting Immune Escape of High- and Low-Risk Groups

**Figure 7:**
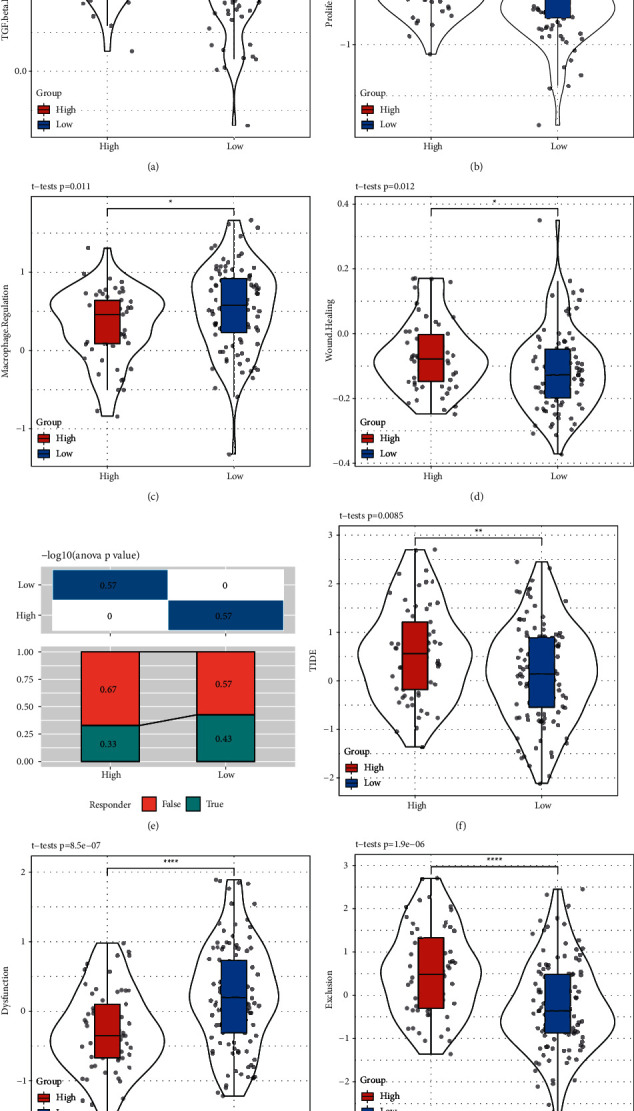
TIDE prediction of the efficiency to immunotherapy in TCGA-PAAD dataset. (a–d) The score of TGF-*β* response (a) proliferation (b) macrophage regulation (c) and wound healing (d) in two groups. Student *t* test was performed. (e) The percentage of positive and negative response to immunotherapy predicated by TIDE in high-risk and low-risk groups. False indicates negative response and true indicates positive response. ANOVA test was performed. (f–h) TIDE score (f), T cell dysfunction score (g) and T cell exclusion (h) score in high-risk and low-risk groups. Student *t* test was performed. ^*∗*^*P* < 0.05, ^*∗∗*^*P* < 0.01, ^*∗∗∗*^*P* < 0.001, ^*∗∗∗∗*^*P* < 0.0001.

We obtained the scores of four immune signatures (TGF-*β* response, proliferation, macrophage regulation, and wound healing) from a previous study using the same TCGA-PAAD dataset [5], and compared their scores in high- and low-risk groups. Higher scores on TGF-*β* response, proliferation, and wound healing were found in the high-risk group while the low-risk group showed higher scores on macrophage regulation (*P* < 0.05, Figures 7(a)–7(d)), which was consistent with the result in the previous section that TGF-*β* signaling pathway was more enriched in high-risk group (Figure 5(c)). The result also indicated that the high-risk group not only had an active inflammatory response but a high proliferation score was presented simultaneously that may override its immune response.

To predict the immune escape if accepting immunotherapy, we used TIDE measurement to evaluate the possibility that patients could benefit from the immunotherapy. The result showed that the low-risk group had a higher proportion (43%) of patients who can benefit much from the immunotherapy than the high-risk group (33%) (Figure 7(e)). A high TIDE score was presented in the high-risk group, suggesting that less benefit from immunotherapy could be obtained (*P*=0.0085, Figure 7(f)). In addition, the low-risk group exhibited higher T cell dysfunction and lower T cell exclusion than the high-risk group (*P* < 0.0001, Figures 7(g) and 7(h)).

Differential response of high- and low-risk groups to immunotherapy and chemotherapy.

**Figure 8:**
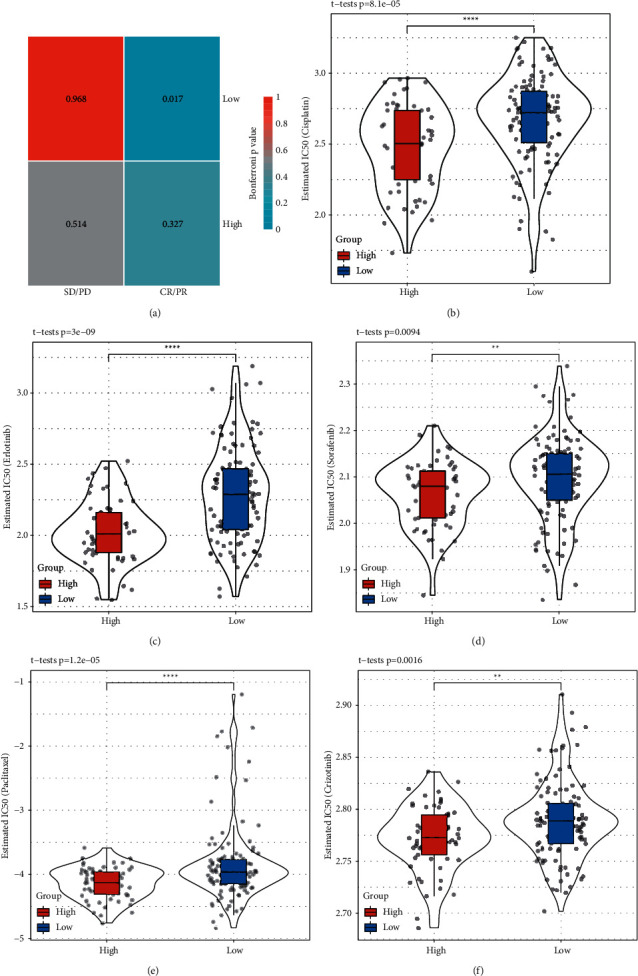
Sensitivity analysis to immunotherapy and chemotherapy in TCGA-PAAD dataset. (a) SubMap analysis between TCGA-PAAD and IMvigor210 datasets. SD/PD indicates the group of stable disease (SD) and progressive disease (PD). CR/PR indicates the group of complete response (CR) and partial response (PR). Chi-square test was performed. (b–f) Comparision of estimated IC50 of cisplatin (b) erlotinib (c), sorafenib (d) paclitaxel (e) and crizotinib (f) between high-risk and low-risk groups. Student *t* test was performed. ^*∗∗*^*P* < 0.01, ^*∗∗∗∗*^*P* < 0.0001.

Finally, we assessed the sensitivity of subtypes to immunotherapy and chemotherapy. We used an IMvigor210 dataset including the treatment data of anti-PD-L1 therapy for metastatic urothelial carcinoma and employed SubMap analysis to compare the similarity of expression data between IMvigor210 and TCGA-PAAD datasets. The result showed that the low-risk group was more similar to CR/PR patients in IMvigor210 than the high-risk group, suggesting a higher sensitivity to anti-PD-L1 therapy (*P* = 0.017, Figure 8(a)). The observation was consistent with the result of the TIDE prediction (Figure 7(f)). Furthermore, we evaluated the estimated IC50 of five chemotherapeutic drugs (cisplatin, erlotinib, sorafenib, paclitaxel, and crizotinib) in low-risk and high-risk groups. We found that the high-risk group had significantly lower estimated IC50 of all five drugs (*P* < 0.01, Figures 8(b)–8(f), indicating that the high-risk group was more sensitive to chemotherapy. Overall, the low-risk group had a superior manifestation in both immunotherapy and chemotherapy.

## 4. Discussion

A TGF-*β* signaling pathway is highly active in PAAD and can function dual roles in suppressing and promoting cancer development. This study focused on the TGF-*β* signaling pathway and constructed a 7-gene prognostic model as a signature based on TGF-*β*-associated genes. This prognostic signature manifested robust performance that can clearly stratify samples into high-risk and low-risk groups in the training group (TCGA-PAAD) and validation group (GSE cohorts and ICGC dataset). Significantly differential prognosis was observed in high-risk and low-risk groups with poor OS in the high-risk group. The risk score is an independent risk factor and presents high HR compared to other risk factors in both univariate and multivariate Cox regression analysis.

Among 7 prognostic genes in the model, SMAD3 and SMAD6 are involved in the Smad pathway that format Smad complex and interact with transcriptional factors, and thus, regulate the expression of TGF-*β* targeted genes [23]. SMAD3 promotes cancer progression by inhibiting natural killer (NK) cells, and SMAD3-silenced NK cells can enhance cancer immunotherapy [24, 25]. CDK9 belongs to a family of CDK enzymes responsible for cell proliferation and development, which has been reported to be associated with cancer progression in many cancer types [26]. It is considered as a therapeutic target in colorectal cancer [27], prostate cancer [28], and also in pancreatic cancer [29]. WWTR1 and TGIF1 were also reported to be involved in cancer development in various cancer types [30, 31].

We analyzed hallmark and KEGG pathways in high-risk and low-risk groups and observed that oncogenic pathways were highly enriched in high-risk groups. Besides the TGF-*β* signaling pathway, Notch signaling, glycolysis, and hypoxia pathways showed high enrichment scores in a high-risk group. Activation of the Notch signaling pathway persists from the early to late stages of PAAD pathogenesis and tumorigenesis [32]. It was found that Notch signaling is necessary for epithelial cytostatic response to TGF-*β*, indicating a cross-link between Notch and TGF-*β* signaling pathways [33]. The glycolysis pathway is active in many cancer types, with increased lactate production and glycolytic enzyme overexpression, especially in PAAD [34]. Hypoxia is one of the hallmarks of solid tumors, and it is considered as a therapeutic target in many cancer types such as lung cancer [35]. In pancreatic cancer, hypoxia is an important driving factor in angiogenesis [36] and can remodel the tumor microenvironment under reactive oxygen species (ROS) driven by hypoxia [37].

ECM-receptor interaction and PI3K-Akt signaling pathways within KEGG pathways were also enriched in the high-risk group. The extracellular matrix (ECM) is the fundamental component in the tumor stromal and supports a solid interaction for cancer migration and metastasis. Preclinical studies have proved promising outcomes in developing drugs targeting ECM in PAAD [38]. A combination of ECM targeted therapy and other therapies such as chemotherapy is a common strategy for treating PAAD in clinical trials [38]. PI3K-Akt signaling is a well-known oncogenic pathway in many cancer types, and it is a popular target for cancer therapy. Molecular drugs targeting PI3K-Akt such as urolithin A are developed for PAAD treatment [39]. These oncogenic pathways are highly enriched in the high-risk group with high expression of TGF-*β*, suggesting that they may have direct or indirect cross-links with TGF-*β* signaling in the progression of PAAD.

To evaluate whether this prognostic signature could provide guidance to immunotherapy, we applied TIDE and SubMap analysis for prediction. TIDE analysis revealed a higher TIDE score or higher immune escape in the high-risk group. Although a lower score of T cell dysfunction meaning lower inhibition from TME on T cell function was presented in the high-risk group, a higher score of T cell exclusion representing lower T cell infiltration in TME was simultaneously shown. TIDE predicted that the low-risk group was more sensitive to immunotherapy than the high-risk group. Furthermore, SubMap also supported this prediction that the low-risk group manifested similar expression characteristics compared with a dataset treated with an-PD-L1 immunotherapy, suggesting the low-risk group had a higher response to immunotherapy.

Estimated IC50 calculation showed that the low-risk group was more sensitive to chemotherapeutic drugs including cisplatin, erlotinib, sorafenib, paclitaxel, and crizotinib. In addition, an assessment of inflammatory signatures concluded that interferon and STAT1 signatures were closely correlated with risk score (correlation coefficient > 0.3). Evidence support that interferon can enhance the sensitivity to chemotherapy such as gemcitabine and interferon therapy is expected to be a potential treatment in pancreatic cancer [40, 41]. STAT1 was suggested as a prognostic biomarker in solid tumors and pancreatic cancer in previous studies [42, 43]. A study proposed that STAT1 could also enhance the sensitivity to gemcitabine in pancreatic cancer [44]. These findings further support the reliability and effectiveness of our classification for PAAD patients based on TGF-*β* signaling.

In summary, this study focused on genes within the TGF-*β* signaling pathway and exploited a 7-gene prognostic signature with robust performance in the independent datasets. The signature can effectively divide PAAD patients into high-risk and low-risk groups with the distinct OS. Differential sensitivity to immunotherapy and chemotherapy was presented between the two groups.

## 5. Conclusions

In conclusion, the prognostic signature further demonstrated the important role of TGF-*β* signaling in PAAD progression and its interaction with other oncogenic pathways. Moreover, the signature can provide guidance for applying personalized therapies to PAAD patients.

## Data Availability

The datasets analyzed in this study are available at GSE57495 (https://www.ncbi.nlm.nih.gov/geo/query/acc.cgi?acc=GSE57495), GSE21501 (https://www.ncbi.nlm.nih.gov/geo/query/acc.cgi?acc=GSE21501), GSE28735 (https://www.ncbi.nlm.nih.gov/geo/query/acc.cgi?acc=GSE28735), GSE62452 (https://www.ncbi.nlm.nih.gov/geo/query/acc.cgi?acc=GSE62452), GSE85916 (https://www.ncbi.nlm.nih.gov/geo/query/acc.cgi?acc=GSE85916), GSE71729 (https://www.ncbi.nlm.nih.gov/geo/query/acc.cgi?acc=GSE71729).

## Supplementary Materials

Supplementary Materials Supplementary Figure S1: ROC curves of the 7-gene prognostic signature in TCGA-PAAD, GSE57495, GSE21501, GSE28735, GSE62452, GSE85916, GSE71729, and ICGC datasets. Supplementary Figure S2: the distribution of risk scores in different clinical features. Kruskal–Wallis test was performed in comparison of four groups and the Wilcoxon test was performed in comparison of two groups. Supplementary Figure S3: the performance of the 7-gene signature in different clinical features including genders (A-B), ages (C-D), T stage (E-F), N stage (G-H), M0 stage (I), and stage I. The performance e (K-L). Supplementary Figure S4: functional analysis of genes related to risk score in TCGA-PAAD dataset. (A) Heatmap of genes significantly related to risk score ranking by risk score. Red indicates positive correlation and blue indicates negative correlation. (B–D) The top 10 enriched terms in biological process (B), cellular component (C), molecular function (D), and KEGG pathways. Dot size represents the number of enriched genes. FDR, false discovery rate. Supplementary Figure S5: assessment of immune microenvironment through ESTIMATE and CIBERSORT. (A–C) Stromal score, immune score, and ESTIMATE score of high-risk and low-risk groups. A Student *t* test was performed. (D) CIBERSORT analysis for describing enrichment of 22 immune cells in high-risk and low-risk groups. A Student *t* test was performed. ns, no significance. ^*∗*^*P* < 0.05,  ^*∗∗*^*P* < 0.01. Supplementary Table S1: the clinical information of eight datasets (TCGA-PAAD, GSE57495, GSE21501, GSE28735, GSE62452, GSE85916, GSE71729, and ICGC).
