# Supplementary figures and images for: Constructing a Novel Prognostic Signature Based on TGF-β Signaling for Personalized Treatment in Pancreatic Adenocarcinoma
Source: J Oncol. 2022 Sep 16;2022:4419119. doi: 10.1155/2022/4419119 (PMC9507654; doi:10.1155/2022/4419119)

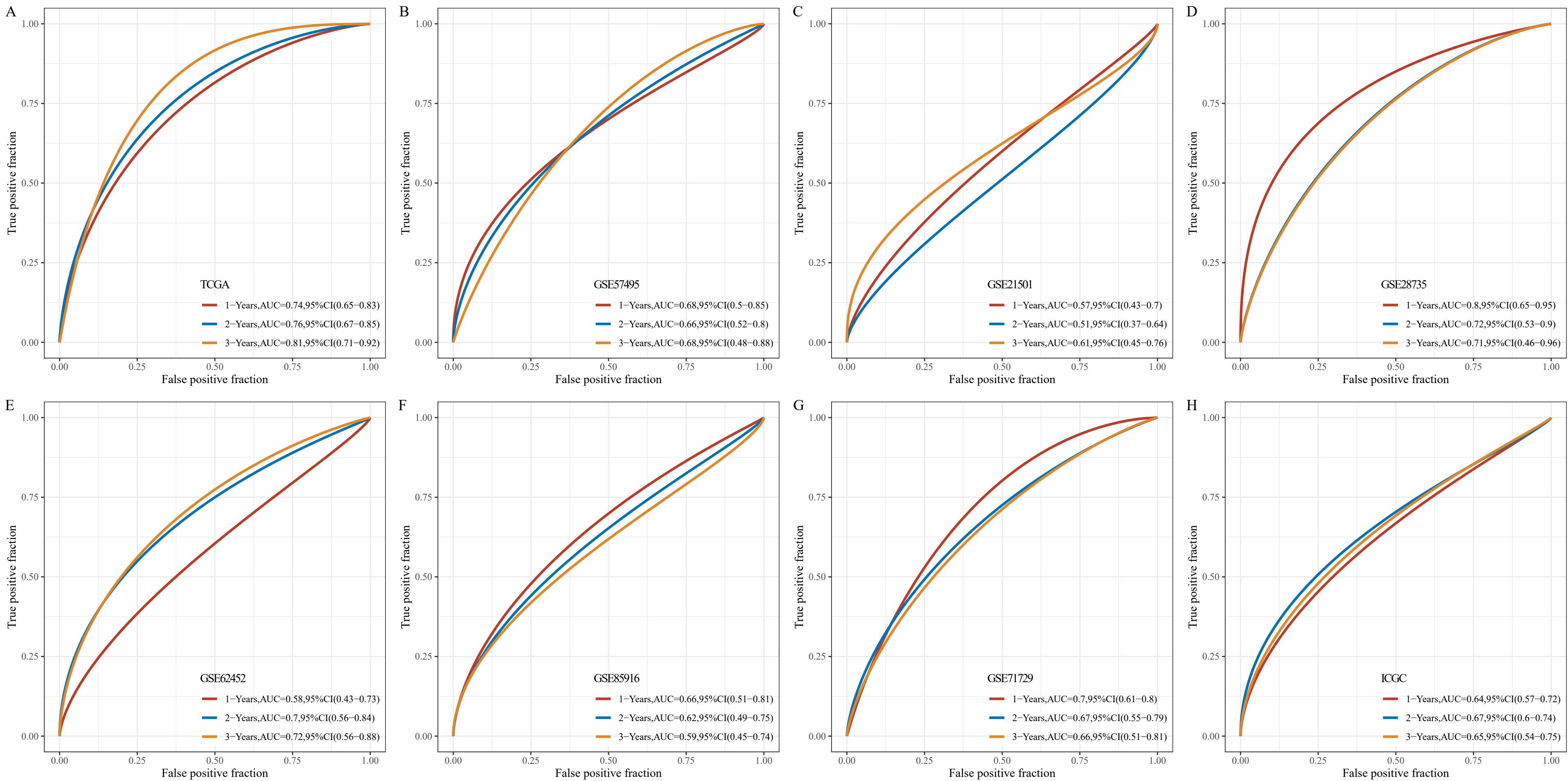

Supplement: Supplementary Materials — Supplementary Figure S1: ROC curves of the 7-gene prognostic signature in TCGA-PAAD, GSE57495, GSE21501, GSE28735, GSE62452, GSE85916, GSE71729, and ICGC datasets. Supplementary Figure S2: the distribution of risk scores in different clinical features. Kruskal–Wallis test was performed in comparison of four groups and the Wilcoxon test was performed in comparison of two groups. Supplementary Figure S3: the performance of the 7-gene signature in different clinical features including genders (A-B), ages (C-D), T stage (E-F), N stage (G-H), M0 stage (I), and stage I. The performance e (K-L). Supplementary Figure S4: functional analysis of genes related to risk score in TCGA-PAAD dataset. (A) Heatmap of genes significantly related to risk score ranking by risk score. Red indicates positive correlation and blue indicates negative correlation. (B–D) The top 10 enriched terms in biological process (B), cellular component (C), molecular function (D), and KEGG pathways. Dot size represents the number of enriched genes. FDR, false discovery rate. Supplementary Figure S5: assessment of immune microenvironment through ESTIMATE and CIBERSORT. (A–C) Stromal score, immune score, and ESTIMATE score of high-risk and low-risk groups. A Student t test was performed. (D) CIBERSORT analysis for describing enrichment of 22 immune cells in high-risk and low-risk groups. A Student t test was performed. ns, no significance. ∗P < 0.05, ∗∗P < 0.01. Supplementary Table S1: the clinical information of eight datasets (TCGA-PAAD, GSE57495, GSE21501, GSE28735, GSE62452, GSE85916, GSE71729, and ICGC). [file 4419119.f1.zip › Supplementary Figure S1 (1).pdf]

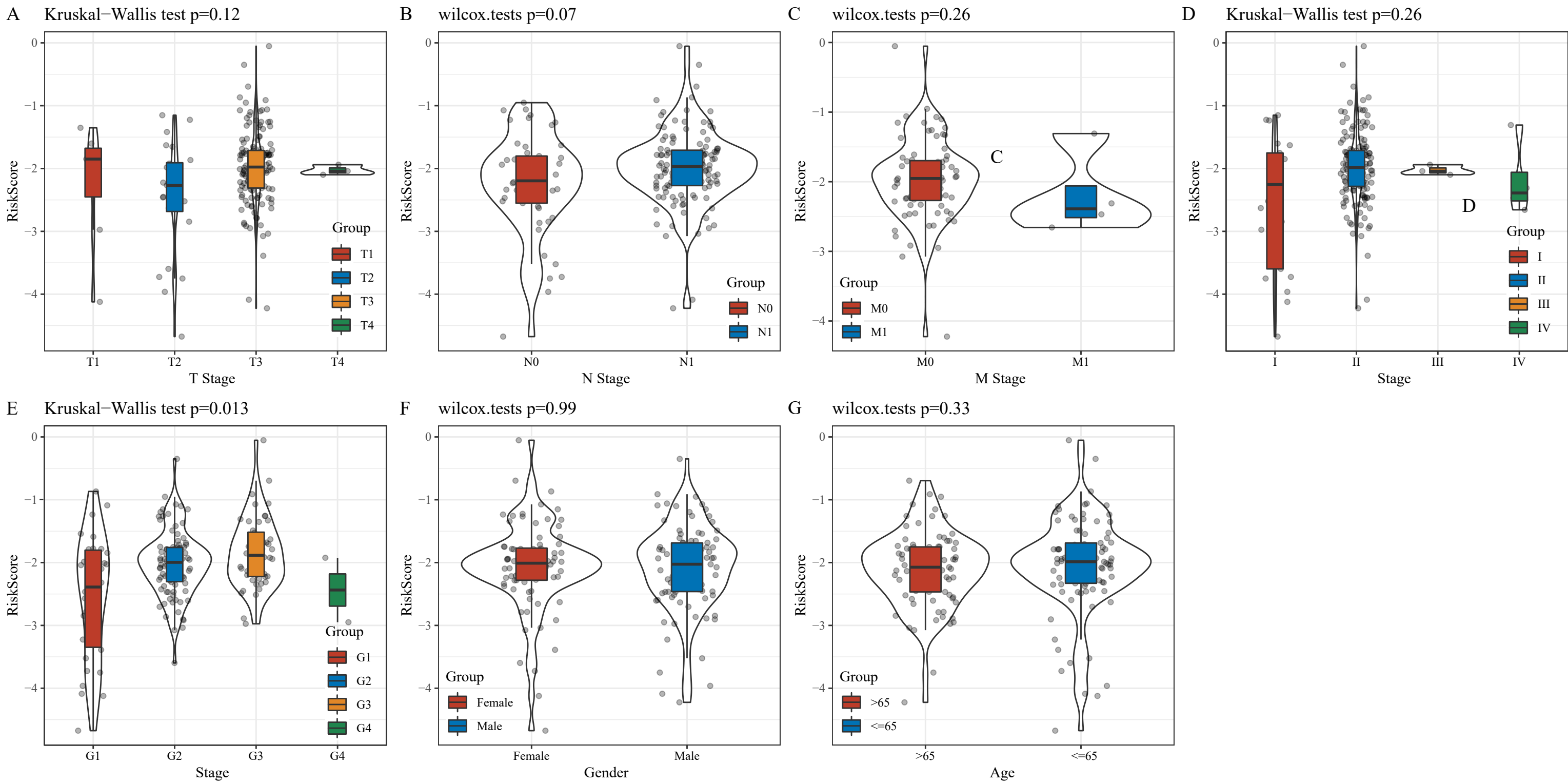

Supplement: Supplementary Materials — Supplementary Figure S1: ROC curves of the 7-gene prognostic signature in TCGA-PAAD, GSE57495, GSE21501, GSE28735, GSE62452, GSE85916, GSE71729, and ICGC datasets. Supplementary Figure S2: the distribution of risk scores in different clinical features. Kruskal–Wallis test was performed in comparison of four groups and the Wilcoxon test was performed in comparison of two groups. Supplementary Figure S3: the performance of the 7-gene signature in different clinical features including genders (A-B), ages (C-D), T stage (E-F), N stage (G-H), M0 stage (I), and stage I. The performance e (K-L). Supplementary Figure S4: functional analysis of genes related to risk score in TCGA-PAAD dataset. (A) Heatmap of genes significantly related to risk score ranking by risk score. Red indicates positive correlation and blue indicates negative correlation. (B–D) The top 10 enriched terms in biological process (B), cellular component (C), molecular function (D), and KEGG pathways. Dot size represents the number of enriched genes. FDR, false discovery rate. Supplementary Figure S5: assessment of immune microenvironment through ESTIMATE and CIBERSORT. (A–C) Stromal score, immune score, and ESTIMATE score of high-risk and low-risk groups. A Student t test was performed. (D) CIBERSORT analysis for describing enrichment of 22 immune cells in high-risk and low-risk groups. A Student t test was performed. ns, no significance. ∗P < 0.05, ∗∗P < 0.01. Supplementary Table S1: the clinical information of eight datasets (TCGA-PAAD, GSE57495, GSE21501, GSE28735, GSE62452, GSE85916, GSE71729, and ICGC). [file 4419119.f1.zip › Supplementary Figure S2 (1).pdf]

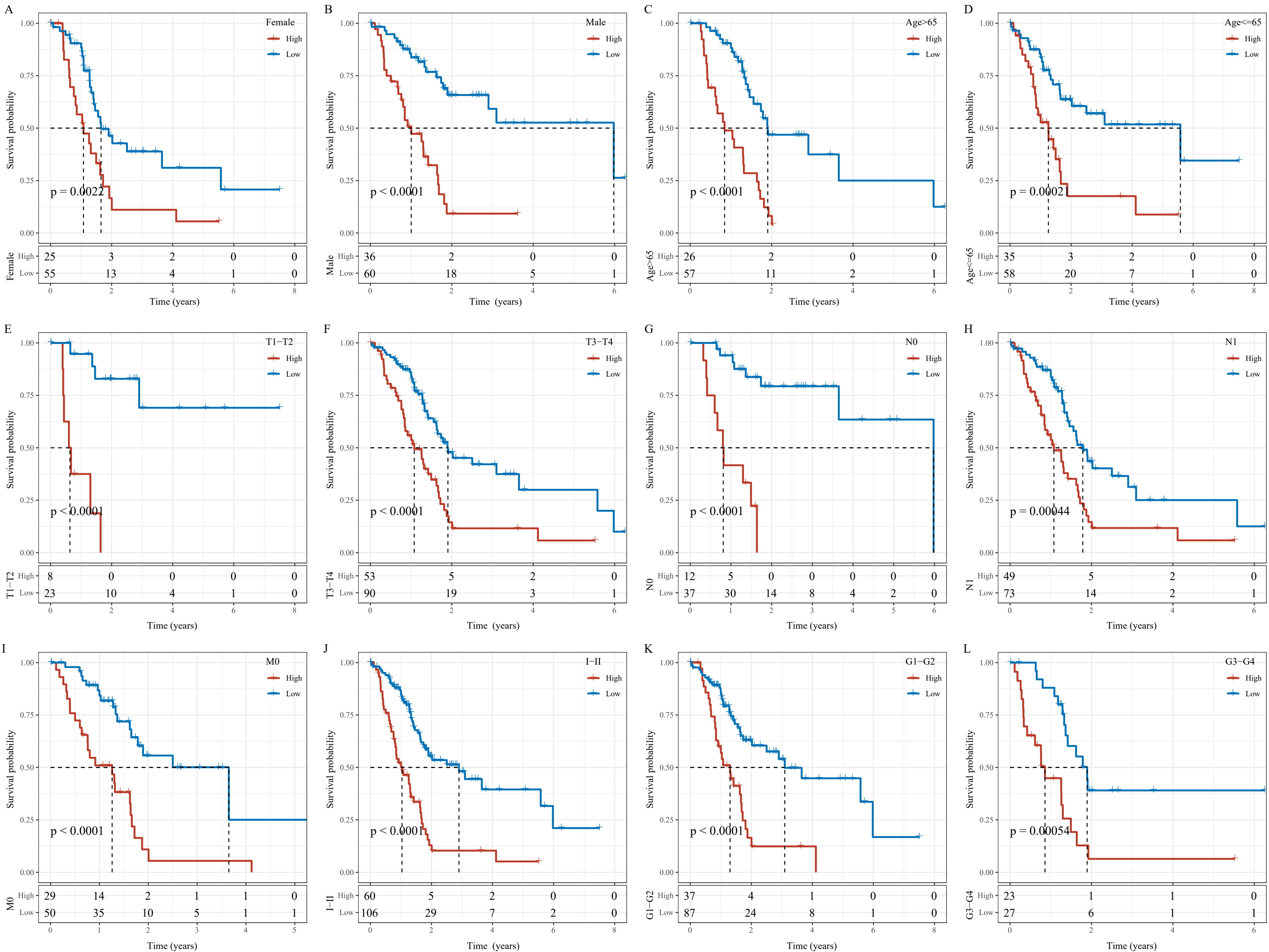

Supplement: Supplementary Materials — Supplementary Figure S1: ROC curves of the 7-gene prognostic signature in TCGA-PAAD, GSE57495, GSE21501, GSE28735, GSE62452, GSE85916, GSE71729, and ICGC datasets. Supplementary Figure S2: the distribution of risk scores in different clinical features. Kruskal–Wallis test was performed in comparison of four groups and the Wilcoxon test was performed in comparison of two groups. Supplementary Figure S3: the performance of the 7-gene signature in different clinical features including genders (A-B), ages (C-D), T stage (E-F), N stage (G-H), M0 stage (I), and stage I. The performance e (K-L). Supplementary Figure S4: functional analysis of genes related to risk score in TCGA-PAAD dataset. (A) Heatmap of genes significantly related to risk score ranking by risk score. Red indicates positive correlation and blue indicates negative correlation. (B–D) The top 10 enriched terms in biological process (B), cellular component (C), molecular function (D), and KEGG pathways. Dot size represents the number of enriched genes. FDR, false discovery rate. Supplementary Figure S5: assessment of immune microenvironment through ESTIMATE and CIBERSORT. (A–C) Stromal score, immune score, and ESTIMATE score of high-risk and low-risk groups. A Student t test was performed. (D) CIBERSORT analysis for describing enrichment of 22 immune cells in high-risk and low-risk groups. A Student t test was performed. ns, no significance. ∗P < 0.05, ∗∗P < 0.01. Supplementary Table S1: the clinical information of eight datasets (TCGA-PAAD, GSE57495, GSE21501, GSE28735, GSE62452, GSE85916, GSE71729, and ICGC). [file 4419119.f1.zip › Supplementary Figure S3 (1).pdf]

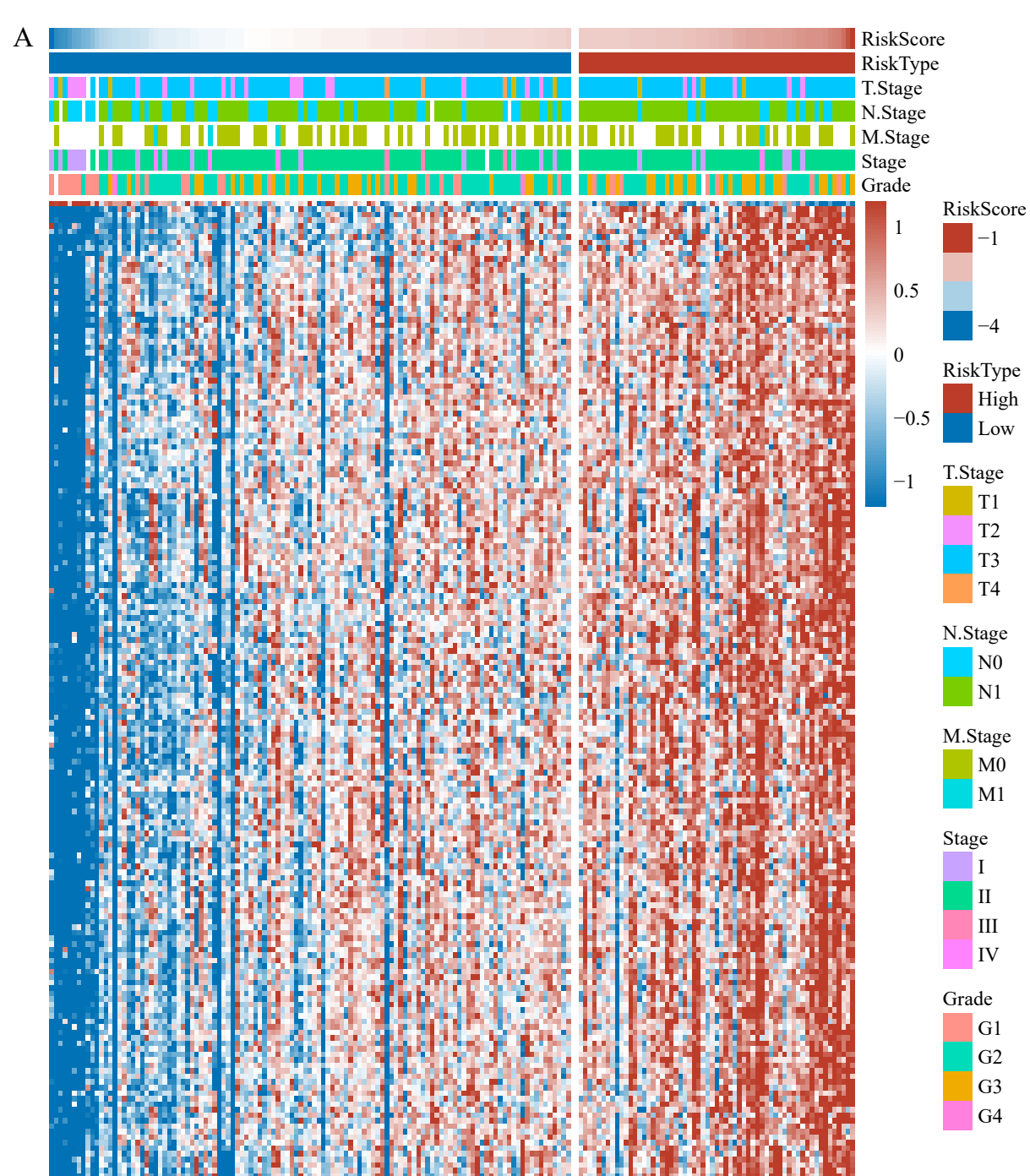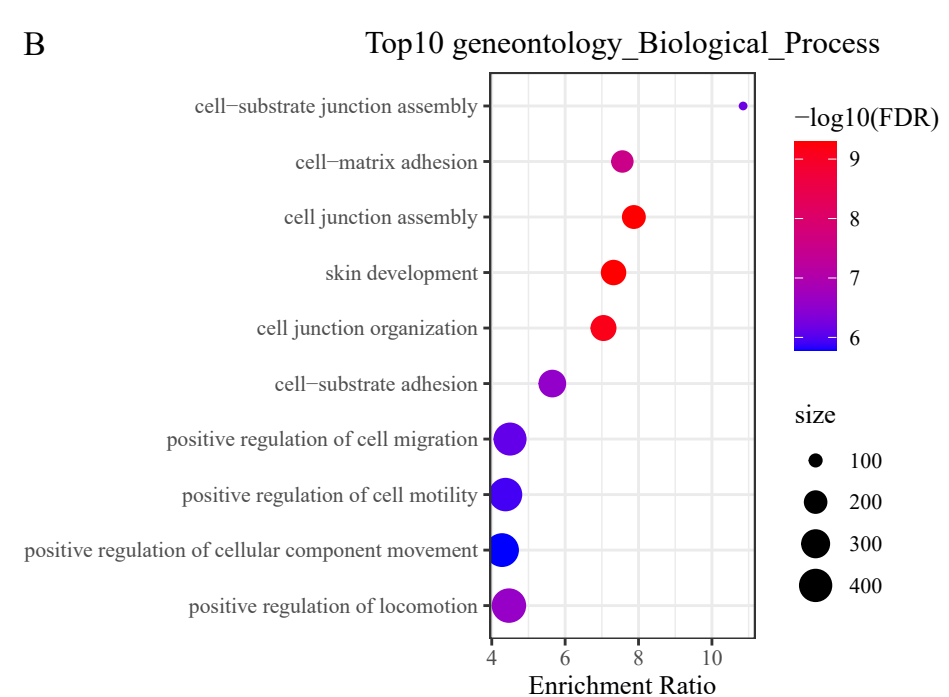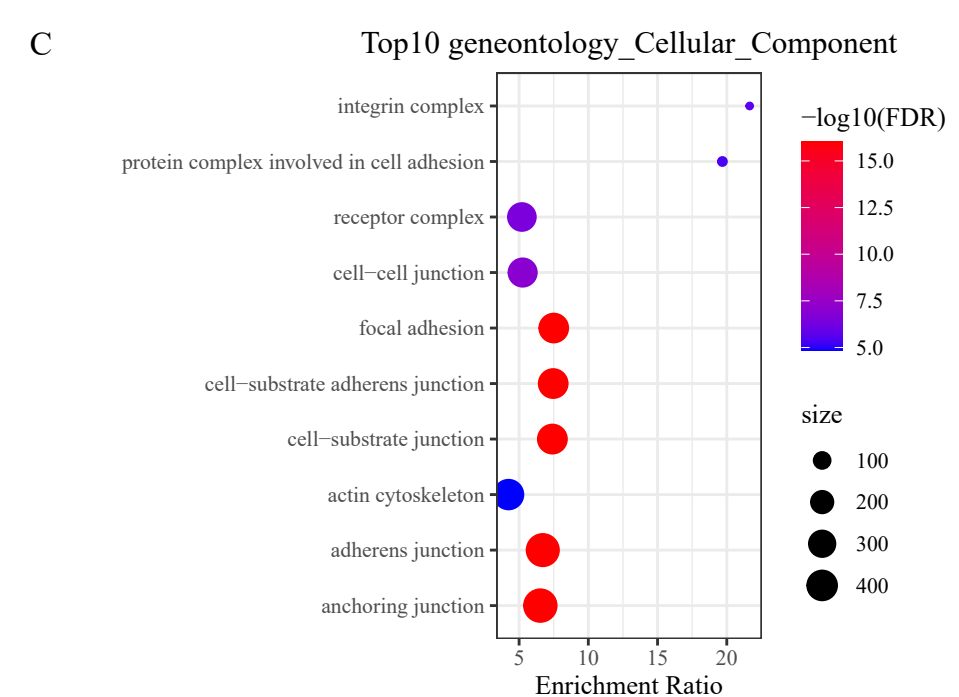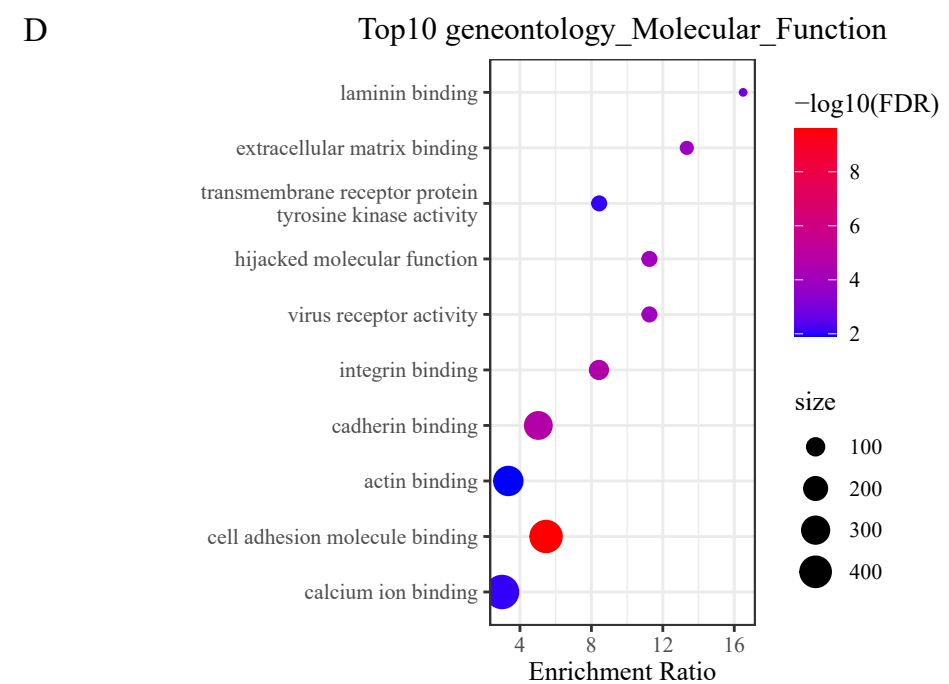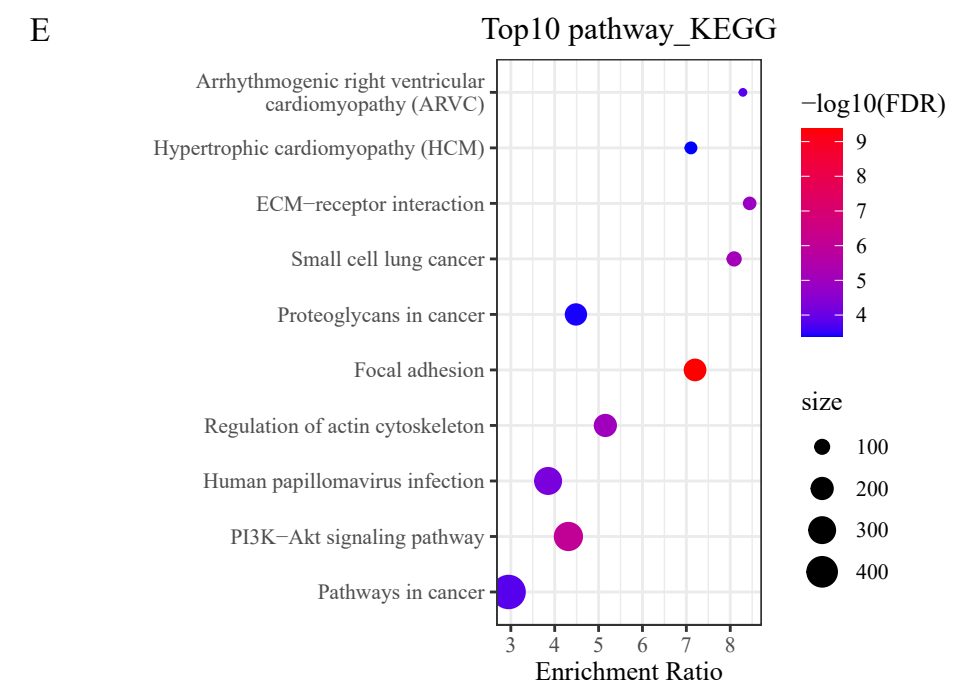

Supplement: Supplementary Materials — Supplementary Figure S1: ROC curves of the 7-gene prognostic signature in TCGA-PAAD, GSE57495, GSE21501, GSE28735, GSE62452, GSE85916, GSE71729, and ICGC datasets. Supplementary Figure S2: the distribution of risk scores in different clinical features. Kruskal–Wallis test was performed in comparison of four groups and the Wilcoxon test was performed in comparison of two groups. Supplementary Figure S3: the performance of the 7-gene signature in different clinical features including genders (A-B), ages (C-D), T stage (E-F), N stage (G-H), M0 stage (I), and stage I. The performance e (K-L). Supplementary Figure S4: functional analysis of genes related to risk score in TCGA-PAAD dataset. (A) Heatmap of genes significantly related to risk score ranking by risk score. Red indicates positive correlation and blue indicates negative correlation. (B–D) The top 10 enriched terms in biological process (B), cellular component (C), molecular function (D), and KEGG pathways. Dot size represents the number of enriched genes. FDR, false discovery rate. Supplementary Figure S5: assessment of immune microenvironment through ESTIMATE and CIBERSORT. (A–C) Stromal score, immune score, and ESTIMATE score of high-risk and low-risk groups. A Student t test was performed. (D) CIBERSORT analysis for describing enrichment of 22 immune cells in high-risk and low-risk groups. A Student t test was performed. ns, no significance. ∗P < 0.05, ∗∗P < 0.01. Supplementary Table S1: the clinical information of eight datasets (TCGA-PAAD, GSE57495, GSE21501, GSE28735, GSE62452, GSE85916, GSE71729, and ICGC). [file 4419119.f1.zip › Supplementary Figure S4.pdf]

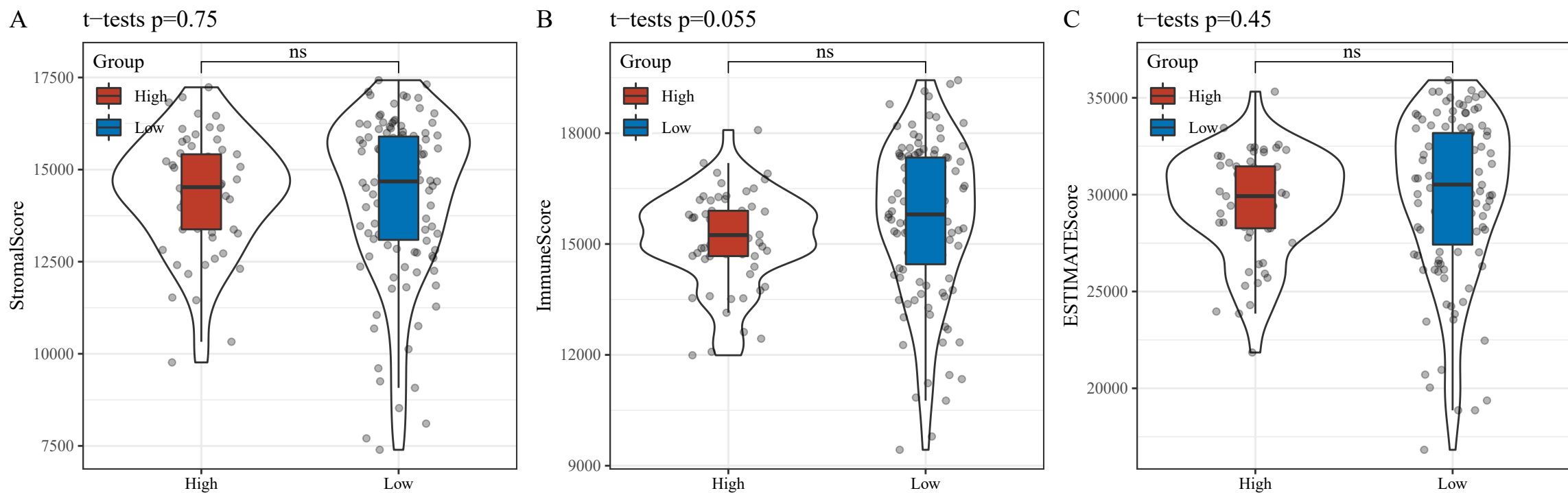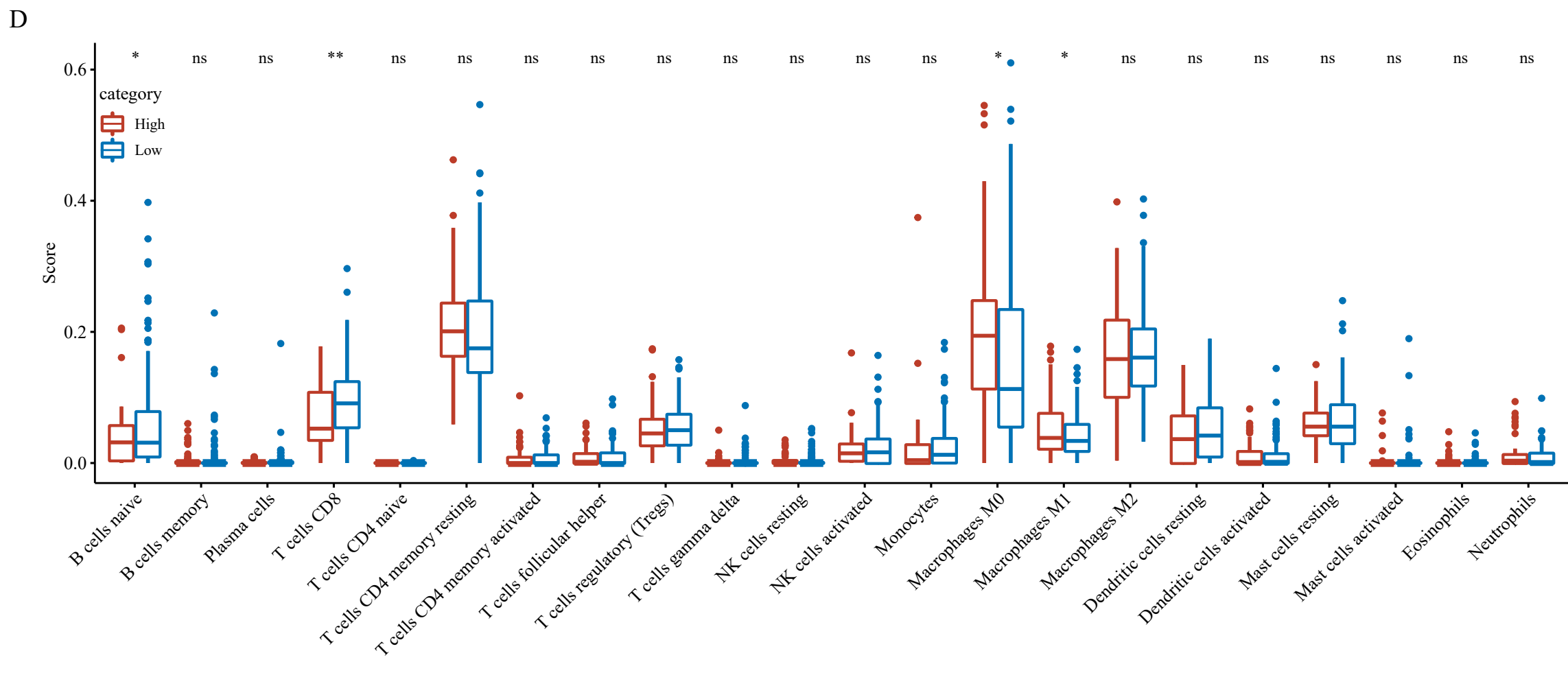

Supplement: Supplementary Materials — Supplementary Figure S1: ROC curves of the 7-gene prognostic signature in TCGA-PAAD, GSE57495, GSE21501, GSE28735, GSE62452, GSE85916, GSE71729, and ICGC datasets. Supplementary Figure S2: the distribution of risk scores in different clinical features. Kruskal–Wallis test was performed in comparison of four groups and the Wilcoxon test was performed in comparison of two groups. Supplementary Figure S3: the performance of the 7-gene signature in different clinical features including genders (A-B), ages (C-D), T stage (E-F), N stage (G-H), M0 stage (I), and stage I. The performance e (K-L). Supplementary Figure S4: functional analysis of genes related to risk score in TCGA-PAAD dataset. (A) Heatmap of genes significantly related to risk score ranking by risk score. Red indicates positive correlation and blue indicates negative correlation. (B–D) The top 10 enriched terms in biological process (B), cellular component (C), molecular function (D), and KEGG pathways. Dot size represents the number of enriched genes. FDR, false discovery rate. Supplementary Figure S5: assessment of immune microenvironment through ESTIMATE and CIBERSORT. (A–C) Stromal score, immune score, and ESTIMATE score of high-risk and low-risk groups. A Student t test was performed. (D) CIBERSORT analysis for describing enrichment of 22 immune cells in high-risk and low-risk groups. A Student t test was performed. ns, no significance. ∗P < 0.05, ∗∗P < 0.01. Supplementary Table S1: the clinical information of eight datasets (TCGA-PAAD, GSE57495, GSE21501, GSE28735, GSE62452, GSE85916, GSE71729, and ICGC). [file 4419119.f1.zip › Supplementary Figure S5.pdf]
